# Establishing *Galleria mellonella* as an invertebrate model for the emerging multi-host pathogen *Helcococcus ovis*

**DOI:** 10.1080/21505594.2023.2186377

**Published:** 2023-03-12

**Authors:** Federico Cunha, Alexandra Burne, Segundo Casaro, Mary B. Brown, Rafael S. Bisinotto, Klibs N. Galvao

**Affiliations:** a Department of Large Animal Clinical Sciences, University of Florida College of Veterinary Medicine, Gainesville, FL, USA; b Department of Animal Sciences, University of Florida College of Agriculture and Life Sciences, Gainesville, FL, USA; c D. H. Barron Reproductive and PerinatalBiology Research Program, University of Florida, Gainesville, FL, USA; d Department of Infectious Diseases and Immunology, University of Florida College of Veterinary Medicine, Gainesville, FL, USA

**Keywords:** *Galleria mellonella*, metritis, *Helcococcus ovis*, infection model, histology, infectious disease

## Abstract

*Helcococcus ovis* (*H. ovis*) can cause disease in a broad range of animal hosts, including humans, and has been described as an emerging bacterial pathogen in bovine metritis, mastitis, and endocarditis. In this study, we developed an infection model that showed *H. ovis* can proliferate in the hemolymph and induce dose-dependent mortality in the invertebrate model organism *Galleria mellonella* (*G. mellonella*). We applied the model and identified *H. ovis* isolates with attenuated virulence originating from the uterus of a healthy post-partum dairy cow (KG38) and hypervirulent isolates (KG37, KG106) originating from the uterus of cows with metritis. Medium virulence isolates were also isolated (KG36, KG104) from the uterus of cows with metritis. A major advantage of this model is that a clear differentiation in induced mortality between *H. ovis* isolates was detected in just 48 h, resulting in an effective infection model able to identify virulence differences between *H. ovis* isolates with a short turnaround time. Histopathology showed *G. mellonella* employs hemocyte-mediated immune responses to *H. ovis* infection, which are analogous to the innate immune response in cows. In summary, *G. mellonella* can be used as an invertebrate infection model for the emerging multi-host pathogen *Helcococcus ovis.*

## Introduction

*Helcococcus ovis* (*H. ovis*), a catalase-negative, Gram-positive, facultatively anaerobic bacterium, is a little-studied multi-host pathogen that often evades clinical detection due to the unique conditions needed for its isolation in culture media. *H. ovis* was first isolated from necropsy samples of lung, liver, and spleen, and from milk associated with subclinical mastitis in domestic sheep [1]. In both cases, the bacterium co-occurred and displayed satellitism with *Trueperella pyogenes* or *Staphylococcus* spp. Satellitism is likely a result of *H. ovis* dependence on pyridoxal, which is provided by the co-occurring bacteria [2]. That same study identified *H. ovis* as an emerging pathogen in bovine endocarditis and noted the case presentations resemble that of endocarditis in humans caused by *Abiotrophia defectiva*, *Granulicatella adiacens*, and *Granulicatella elegans* [2]. *H. ovis* itself has been identified by 16S rRNA gene sequencing from 19 different human body sites and was associated with cases of peritoneal effusion, vaginal infections, and endometrial neoplasms [3]. Given *H. ovis’* recurrent appearance in mixed infections and its apparent reliance on co-occurring bacteria for pyridoxal metabolism, its ability to independently cause extravascular infections is not well established. Co-isolation with other known pathogenic bacteria continued to be a feature of *H. ovis* isolates from infections in bovine hosts including metritis, mastitis, hepatic abscesses, and septic arthritis [4–7].

Although *H. ovis* is present in the uterus of healthy cows, a higher abundance of this bacterium has been associated with metritis diagnosis [8]. Bovine metritis, the infection and inflammation of the uterus, is a highly prevalent and costly post-partum disease in dairy cows [9,10]. Metritis is associated with dysbiosis of the uterine microbiota [11]; therefore, given the complexity of the uterine microbiota, it is still unclear what role *H. ovis* plays in the pathogenesis of this disease. An *in vivo* murine model for *H. ovis* infection of the mammary gland has recently been established; therefore, a murine model for *H. ovis* infection of the uterus could also be established [5]. However, vertebrate models are expensive and raise ethical concerns.

The larvae of the greater wax moth (*Galleria mellonella*) have been used as an alternate infection model for several bacterial pathogens affecting domestic mammals and humans including *Listeria monocytogenes, Streptococcus agalactiae*, and *Mycobacterium bovis* [12–14]. Their wide availability, low cost, and handling ease provide an excellent tool for high throughput screening of isolates and mutants while fulfilling a substantial role in reducing, replacing, and refining animal use in research. The model is particularly useful as an alternative to mice and other vertebrate hosts in these early stages of *H. ovis* research where high throughput screening of isolates is necessary to begin exploration of a bacterium’s pathogenic potential. Furthermore, since *G. mellonella* larvae rely exclusively on an innate immune response to infection and do not possess an adaptive immune system, they have the potential to become a useful model organism for bovine metritis, as the predominant bovine response to metritis is driven by the innate immune response.

Therefore, in this study, we established the use of *G. mellonella* larvae as an in vivo model for *H. ovis* infection. We identified *H. ovis* isolates with high, medium, and low virulence, and observed that *H. ovis* proliferated in the hemolymph and induced dose-dependent mortality with a quick turnaround of only 48 h. Finally, we showed *G. mellonella* employed hemocyte-mediated immune responses which are analogous to the innate immune response in cows.

## Results

### G.mellonella *larvae are susceptible to* H. ovis *infection*

**Figure 1.**
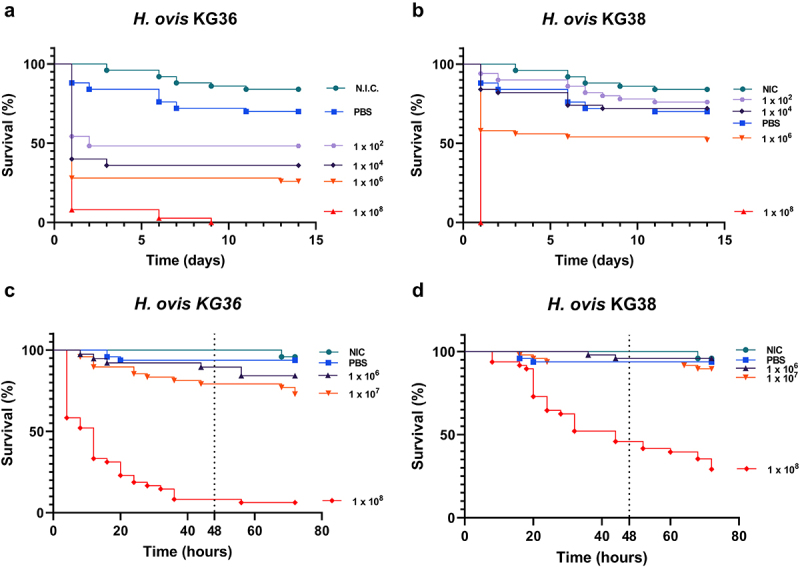
Dose-dependent virulence of *Helcococcus ovis* isolates incubated at 37°C for 14 d (KG36 [a] and KG38 [b]) or at 36°C for 72 h (KG36 [c] and KG38 [d]). The survival data are plotted using the Kaplan–Meier estimator. The plotted points represent mortality events. The dotted line represents the 48-h mark used as end point for log-rank survival analysis.

We tested the susceptibility *G. mellonella* larvae to *H. ovis* infection by two different isolates at infective doses ranging from 1 × 10^2^ to 1 × 10^8^ CFU as a first step to assess the suitability of the infection model. *H. ovis* KG36 originates from the uterus of a cow with current active metritis. *H. ovis* KG38 originates from the uterus of a healthy post-partum cow that did not develop uterine disease. Detailed information on the source of these isolates is included in the supplementary material. The larvae were incubated at 37°C and survival was monitored every 24 h for 14 d. Both isolates induced a dose-dependent killing response as shown in Figure 1(a,b). Both isolates induced 100% mortality within 24 h when injected at 1 × 10^8^ CFU and incubated at 37°C. At the lower dose of 1 × 10^6^, most of the mortality events occurred within the first 3 d of incubation suggesting incubation periods could be significantly shortened. Finally, culture filtrates of KG36 and KG38 were injected into two groups of larvae to rule out an effect of potential suspended metabolites or exotoxins. No significant mortality was observed in the filtrate injection groups when compared to the brain heart infusion (BHI) injection and to the no injection control (NIC) groups (data not shown).

Although a dose-dependent killing response was observed in the first experiment of this study at 37°C (Figure 1a,b) the observed mortality differences between isolates at the tested infection doses were not ideal for virulence comparisons. There have been reported cases, like that of *Klebsiella pneumoniae*, where although the pathogen is capable of proliferating in hemolymph and inducing mortality in *G. mellonella*, the bioassay is unable to distinguish between hypervirulent and the less virulent common strain [15]. Since in both tested isolates (KG36 and KG38) a large difference in mortality was found between the 1 × 10^6^ CFU and 1 × 10^8^ CFU sequential infective doses, a second experiment using these two doses and an intermediate 1 × 10^7^ CFU dose was carried out to optimize the bioassay. Furthermore, the control groups in this first experiment displayed above expected melanization and mortality when incubated at 37°C. This was likely due to the larvae being incubated for several days the maximum reported survivable ambient temperature for the species. Therefore, subsequent experimental temperatures were adjusted to 36°C, which resulted in an almost complete reduction of morbidity and mortality in the control groups. The second round of inoculations was carried out with infective doses of 1 × 10^6^, 1 × 10^7^, and 1 × 10^8^ CFU to improve the ability to differentiate virulence between isolates. Based on the timing of mortality events observed on the first round of infections, the assay was further improved by narrowing the monitoring window to 72 h and increasing the monitoring frequency. Lowering the incubation temperature to 36°C achieved almost a 0% mortality in the control groups while maintaining dose-dependent killing of larvae by both KG36 and KG38 at the tested infective doses. Injection with 1 × 10^7^ CFU produced a statistically significant difference in mortality in the Kaplan–Meier survival curves between the two isolates (KG36 and KG38) after 48 h of incubation (*p *= 0.0459, Log-rank test).

## Time-lapse monitoring

We used time-lapse photography to monitor the larvae for melanization and movement every hour without disturbance during incubation. Using *G. mellonella* as an infection model offers the ability to use large sample sizes for screening bacterial isolates with relatively low labor input. A primary limiting factor for monitoring frequency after inoculation is work time associated with counting and handling of the larvae to measure movement and color change. Implementing monitoring via time-lapse photography with inexpensive commercially available tools allowed for the simultaneous monitoring of 360 larvae in 1-h intervals for up to 72 h (Figure 1c-d). A clear differentiation in induced mortality between *H. ovis* isolates was detected via time-lapse photography in just 48 h (Figure 1c-d). The final optimized bioassay resulted in an effective invertebrate infection model with the ability to identify virulence differences between the two tested *H. ovis* isolates.

### H. ovis *rapidly proliferates in* G. mellonella *hemolymph*

**Figure 2.**
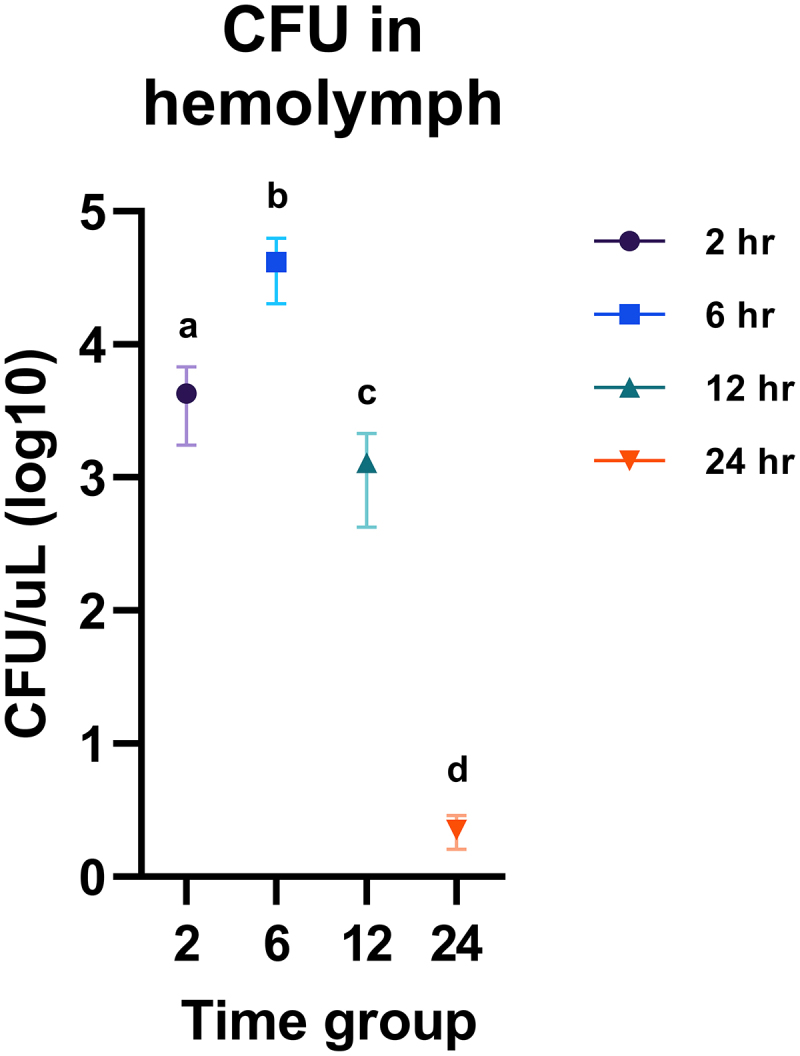
Fecundity measures of bacterial proliferation and survival in hemolymph recovered from infected *G. mellonella*. Raw CFU numbers were transformed to their base-10 logarithm for better visualization. One-way ANOVA followed by multiple comparisons test with Tukey’s correction for multiple comparisons showed all 4 time points had significantly different *H. ovis* CFU in hemolymph. Each lowercase letter (a–d) denotes statistically significant differences in recovered CFU between the time-groups. CFU numbers were similar between KG36 and KG38 isolates at each time point.

**Figure 3.**
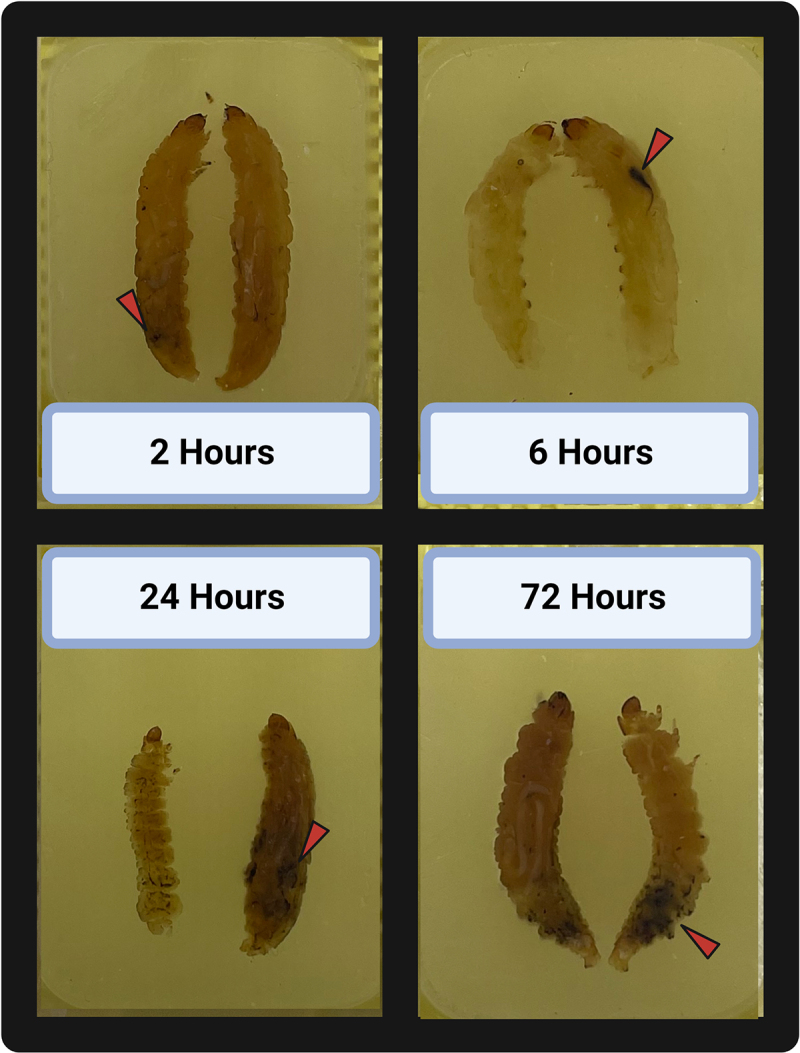
Inoculated *Galleria mellonella* specimens embedded in paraffin, sectioned across the sagittal plane, and laid side by side. Each specimen represents the gross pathology observed in their corresponding incubation time groups. Red triangles point to areas of melanization associated with *H. ovis* infection.

To further verify that the observed mortality in response to *H. ovis* inoculation was a result of bacterial proliferation, we measured bacterial fecundity in hemolymph after injection with 1 × 10^6^ CFU of each isolate via quantitative culture. A common approach to measure bacterial growth in *G. mellonella* is to mechanically homogenize the larvae and to assess the resulting suspension with quantitative culture methods. This approach is not ideal when trying to measure *H. ovis* growth because the normal gut microbiota of *G. mellonella* is dominated by *Enterococci* which can readily grow in Helcococcus Selective Agar and overwhelm the slower growing *H. ovis* 16 Therefore, hemolymph had to be carefully extracted from the larval hemocoel without puncturing the gut to allow *H. ovis* to grow unimpeded. Because these hemolymph extractions were terminal procedures, hemolymph CFU measurements of a time group are independent from the other time groups and do not represent continuous measures of bacterial growth within the same larvae. As shown in Supplemental Figure 3, this measure of proliferation and survival in hemolymph displayed a similar trajectory of bacterial growth and neutralization for both isolates (KG36 and KG38). We first compared KG36 and KG38 at each time point; then, since no difference was found between strains, we aggregated the data and compared each time point against each other. There were three larvae per KG strain; therefore, the time point comparison had six larvae in each group. The central goal in this experiment was to measure whether *H. ovis* are capable of proliferating in the larval hemocoel, which we were able to observe between the 2-h and 6-h groups. Hemolymph CFU reached a mean statistically significant (*p* < 0.0001) peak of 4 × 10^4^ CFU per µL after 6 h of incubation. The larvae evaluated past 12 h showed a significantly lower CFU in hemolymph than the 6-h incubation group (*p* < 0.0001), and even a significantly lower than the levels found in the 2-h incubation group (*p* = 0.0151). Finally, no live *H. ovis* of either isolate was recovered from the group of larvae that survived the entire 24 h of incubation after infection as shown in Figure 2. As illustrated in Figure 3, all surviving larvae showed signs of melanization and nodulation to different degrees. This confirmed that *H. ovis* rapidly replicates in *G. mellonella* hemolymph, and that the larvae can mount an immune response leading to neutralization of the pathogen in surviving larvae.

### G. mellonellaemploy hemocyte-mediated immune responses when infected with H. ovis

**Figure 4.**
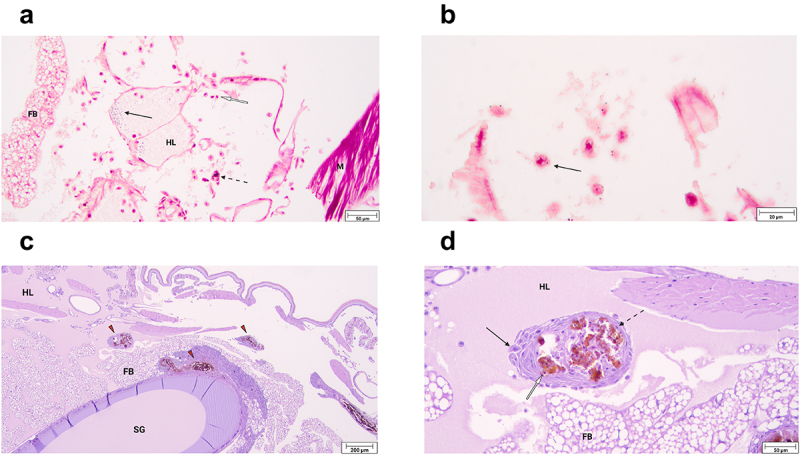
a. Histological slide of *G. mellonella* larvae infected with 1 × 10^7^ CFU of *H. ovis* stained with Gram-stain. Black arrows indicate the presence of large clusters of Gram-positive cocci, white arrows indicate hemocytes recruited to the infection site, and dashed arrows indicate small solitary melanin deposits. Abbreviations: FB, fat body; HL, hemolymph; M, muscle. b. Histological slides of *G. mellonella* larvae infected with 1 × 10^7^ CFU of *H. ovis* stained with Gram-stain. Black arrow indicates a plasmatocyte containing a Gram-positive coccus within its cytoplasm. c-d. Histological slide of *G. mellonella* larvae infected with 1 × 10^7^ CFU of *H. ovis* stained with Hematoxylin and eosin stain. Red triangles indicate mature nodules formed 12 h after infection adhered to the larval fat body and silk gland ©. A magnified view of one of these nodules (d) shows the anatomical composition of these structures characterized by a melanized core encapsulating Gram-positive cocci, an inner layer of hemocytes with melanin inclusions (white arrow), a middle layer of flatted hemocytes forming the nodule’s capsule (dotted arrow), and an exterior layer of newly attached cells (black arrow). Abbreviations: FB, fat body; HL, hemolymph; SG, silk gland. .

To identify the major immune response mechanisms employed by *G. mellonella* in response to *H. ovis* infection, we prepared histological slides of randomly selected live larvae at 2, 6, and 12 h of incubation after inoculation with isolate KG36. Large clusters of Gram-positive cocci were observed near the injection site in association with damaged tissue, recruitment of hemocytes, and small solitary melanin deposits (Figure 4A). Evidence of the immediate cellular immune response was observed in hemocytes of varying morphology containing intracellular Gram-positive cocci both at 2 and 6 h of incubation. The hemolymph of infected larvae appears to have more numerous hemocytes associated with bacterial clusters than what is found in uninfected larvae, in which hemocytes are more often associated with fat bodies. Figure 4B shows hemocytes responding to the cocci still present in the hemocoel 2 h after inoculation. A plasmatocyte containing a Gram-positive coccus within its cytoplasm can be observed, and in this context provides circumstantial evidence of endocytosis. Signs of humoral encapsulation of bacteria via hemolymph coagulation and melanin deposition were observed throughout the hemocoel of all the infected larvae. These areas are easily identified by the dark melanin obscuring all other underlying features, including any encapsulated Gram-positive cocci (Supplemental Figure 4a). The humoral capsules shown are in the early stages of nodule formation which completely mature 12–24 h post-infection. Figure 4c shows representative examples of mature nodule formation adhered to the silk gland and the fat body of the specimen in a 24-h incubated larva. A magnified view of one of these nodules (Figure 4d) shows the classic anatomical composition of these structures characterized by a melanized core encapsulating Gram-positive cocci, an inner layer of hemocytes with melanin inclusions, a middle layer of flatted hemocytes forming the nodule’s capsule, and an exterior layer of newly attached cells. Together, these histological images show *G. mellonella* is capable of successfully deploying both humoral and cellular components of the innate immune system to neutralize *H. ovis*.

### H. ovis isolate from a healthy cow shows comparatively attenuated virulence

**Figure 5.**
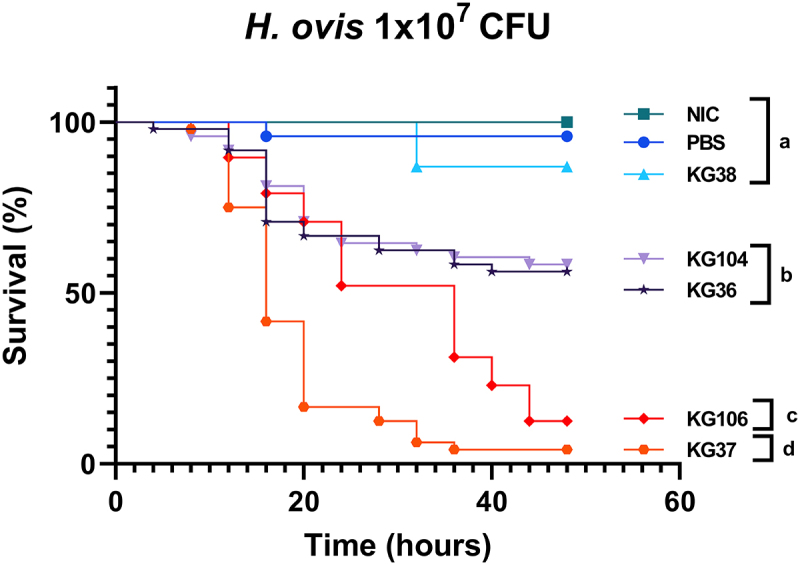
Survival rate of *G. mellonella* larvae injected with 1 × 10^7^ CFU of *H. ovis* isolates KG36, KG37, KG38, KG104, and KG106 and incubated for 48 h at 36°C. Survival data were plotted using the Kaplan–Meier method and comparisons between groups were made using the log-rank test with a Bonferroni adjusted *p-*value for multiple comparisons of *p <* 0.001. Each lowercase letter (a–d) denotes statistically significant differences between the Kaplan–Meier survival curves. Curves labeled with the same letter were not statistically significantly different from each other.

To further test the ability of this model to detect differences in virulence between *H. ovis* isolates, three more *H. ovis* isolates (KG37, KG104, and KG106) originating from the uterus of cows with metritis were tested alongside the previously used ones (KG36 and KG38) using the optimized parameters. The Kaplan–Meier survival analysis shows three distinct groups of mortality caused by the tested isolates found to be statistically significant from each other on Kaplan–Meier survival analysis with a Bonferroni adjusted *p* value for multiple comparisons of *p <* 0.001. There was no significant difference in the Kaplan–Meier survival curves between KG38 and the PBS control group (*p =* 0.077, log-rank test). The Kaplan–Meier survival curves for both KG36 and KG104 were not different from each other (*p =* 0.7970, log-rank test) but were significantly different from all other groups (p *<*0.0005, log-rank test). The Kaplan–Meier survival curves for both KG37 and KG106 were significantly different from each other (*p* < 0.0001, log-rank test) and significantly different from all other groups (p *<0.0001*, log-rank test). The difference between these two highly virulent groups is primarily due to KG37 causing rapid mortality in the first 24 h before both strains converging at a survival probability of less than 15% at 48 h. As shown in Figure 5, this resulted in two highly virulent isolates (KG106 and KG37), two isolates with relatively mild virulence (KG36 and KG104) and one isolate with attenuated virulence (KG38). The *H. ovis* isolate originating from a healthy cow (KG38) caused a significantly lower mortality than all other tested isolates originating from cows with metritis.

## Discussion

In this study, we showed that *H. ovis* isolated from the uterus of dairy cows can establish an active infection in *G. mellonella* larvae. A previous attempt at establishing a *G. mellonella* infection model for a metritis-causing pathogen of horses, *Taylorella equigenitalis (T. equigenitalis)*, found the bacterium was unable to replicate within this model host [17]. Therefore, the first step in establishing this model was to measure the ability of *H. ovis* to replicate within the larvae and cause disease. We showed via quantitative culture and histopathology that, unlike *T. equigenitalis*, all tested *H. ovis* isolates rapidly replicate in hemolymph and most caused some mortality in *G. mellonella*. A likely explanation is for the differences observed between these bacterial species is that, unlike *H. ovis*, *T. equigenitalis* is highly adapted to the equine host and is not known to naturally produce disease in other species or body sites. In contrast, *H. ovis* can colonize and cause disease across both species and body sites in an opportunistic fashion. In this study, *H. ovis* was observed through histopathology in a planktonic state freely disseminated throughout the larval hemocoel or in association with responding hemocytes; no bacterial aggregations were observed in association with muscle, fat bodies, gut, or silk glands. Significant and rapid larval mortality was observed at 1 × 10^7^ CFU by the four *H. ovis* isolates from cows with metritis. Similarly, bovine-associated isolates of *Streptococcus agalactiae*, a multi-host Gram-positive cocci often found in mixed infections with *H. ovis*, have LD_50_ values in *G. mellonella* ranging from 1 × 10^6^ to 1 × 10^7^ CFU [14]. Intramammary injections in mice of mastitis-derived *H. ovis* isolates can cause tissue necrosis at 1 × 10^4^ CFU, but lethal doses in mammalian hosts have yet to be measured [5]. There have been cases where bacterial pathogens induce mortality in *G. mellonella*, but well-established hypervirulent strains of the same species were not able to be differentiated from classical strains [15]. Therefore, using a mammalian model host to corroborate the differential virulence patterns found in *H. ovis* isolates with this *G. mellonella* model would serve as convincing corroboration of this bioassay’s adequacy for virulence prediction in the original host.

The *H. ovis* isolates evaluated in this study all originated from the post-partum uterus of dairy cows with and without uterine disease. The establishment of this bioassay will not only provide insights into the pathogenesis of *H. ovis* infections in other hosts and body sites, but it will also serve as a first step towards achieving an invertebrate model for studying the bacterial pathogenesis of metritis in dairy cows. Co-infection of murine mammary glands with *H. ovis* and *T. pyogenes* has been shown to cause more severe disease than individual infections, which shows *H. ovis* has the capacity to synergize with co-infecting pathogens [5]. *Fusobacterium necrophorum (F. necrophorum), Bacteroides pyogenes*, and *Porphyromonas levii* are other pathogenic bacteria that, in mixed infections with *H. ovis*, have been associated with metritis in dairy cows [8]. Synergistic effects between *F. necrophorum* and other bacterial pathogens in the context of uterine infection have been proposed and the mechanisms have been evaluated ex-vivo [18]. However, the interactions among these four pathogens in the context of a responding immune system have never been evaluated *in vivo*. Studies that have established *G. mellonella* infection models for closely related species in the genus *Fusobacteria*, *Bacteroides*, and *Porphyromonas* are an indication that the use of this invertebrate model to study the bacterial pathogenesis of metritis is likely achievable [19,20].

In this study we used histopathology of infected larvae to evaluate the similarities between the reported bovine host uterine immune response to that of the larvae in the face of *H. ovis* infection. The immune response to bacterial infection in the uterus of dairy cows is primarily driven by the innate immune system. The *G. mellonella* immune system is exclusively comprised of humoral and cellular responses analogous to those found in the bovine response to metritis. As shown in Figures 3 and 4d, large melanin aggregates are present in the histological samples of all inoculated larvae as a response to *H. ovis* infection. The initial response to neutralize microbial invasion both in the post-partum uterus of dairy cows and in the hemocoel of *G. mellonella* is carried out by antimicrobial peptides and humoral immune responses. For example, upon recognition of pathogen-associated molecular patterns, bovine uterine epithelial cells express β-defensins like lingual antimicrobial peptide and tracheal antimicrobial peptide [21]. Gallerimycin is a major antimicrobial peptide in *G. mellonella* with structural and functional similarities to human and mouse β-defensins and is released into the hemolymph in response to detection of bacteria by the larval fat body [19]. Another major non-specific humoral response found in the endometrium of post-partum dairy cows is the upregulation of genes associated with complement system activation, which leads to the assembly of cytotoxic and opsonic complexes to lyse bacterial cells [22]. *G. mellonella* uses a similarly regulated prophenoloxidase activating system, with analogous intermediary steps to the complement cascade in mammals, that culminates in the deposition of melanin which serves as an opsonin and cytotoxin to the bacterial cell. Both melanin and the component system feed into activation of the hemolymph and blood coagulation cascades respectively [23]. Polymorphonuclear neutrophils (PMNs) recruited out of circulation into the endometrium comprise the bulk of the cellular immune response to metritis in the uterus of postpartum dairy cows. Similarly, we showed hemocytes recruited into the larval hemocoel congregate around and interact with the invading cocci (Figure 4a,b). Although we did not evaluate specific hemocyte cellular responses using markers of phagocytosis, *G. mellonella* hemocytes have been shown to respond to infection by Gram-positive bacteria employing phagocytosis [24]. Further, organisms of the genus Helcococcus are not known to be capable of intracellular infection of host cells. Therefore, the presence of *H. ovis* within the cytoplasm of responding hemocytes suggests these bacteria have been endocytosed and are in the process of being neutralized. Nonetheless, further studies are needed to elucidate the cellular interactions between *H. ovis* and responding *G. mellonella* hemocytes. Hemocytes of *G. mellonella* perform all the same functions as bovine PMNs including lectin-mediated phagocytosis, reactive oxygen species production, degranulation, and the extrusion of extracellular nets and DNA for bacterial killing [25]. Together, these observed larval immune mechanisms suggest the G. mellonella infection model can provide suitable in-vivo analogue to the immune to environment endured by bacterial pathogens in the bovine uterus. Although nodule formation by the invertebrate model organism is not an immune response with a direct analogue in the context of metritis, it does occur in *G. mellonella* as a final step after a successful incapacitation of the invading pathogens with antimicrobial peptides and phagocytic cell action [26]. The cellular anatomy of the *G. mellonella* nodules observed in this study (Figure 4e-f) has a remarkably similar cellular arrangement to that of abscesses formed in response to bacterial infection in mammals. This response can serve as an analogue for the widely reported abscess formation in cases of *H. ovis* infection of lungs, liver, spleen, and other organs across other domestic animal hosts.

Time-lapse photography to monitor larval mortality enabled us to increase throughput, reduce handling of larvae, and increase the frequency of time intervals in the survival curve. The sample size to establish the infection model was calculated to detect a 25% difference in mortality between isolates. However, in this experiment, we observed differences in induced mortality between *H. ovis* strains of 30% or greater. These established larval survival differences would allow us to reduce the number of larvae used to 37 per group while maintaining a significance level of 5% and power of 80% in future experiments. Further reducing the sample size to 24 larvae per group would be sufficient to detect mortality differences between highly virulent strains and other less virulent or avirulent isolates. However, it would not suffice to differentiate medium virulence strains like KG36 from attenuated ones like KG38.

To confirm the utility of the optimized infection model, we applied it to evaluate the virulence of five *H. ovis isolates* isolated from the uterus of dairy cows. Most interestingly, we found that the isolate (KG38) sourced from the uterus of a dairy cow that never developed post-partum uterine disease displayed attenuated virulence in comparison to the 4 other isolates originating from cows with metritis. Further testing of isolates recovered from cows that do not develop uterine disease should be pursued to assess whether there are *H. ovis* strains with attenuated virulence associated with uterine health. A mammalian infection model, for example, mouse infection model, would be applied to demonstrate this link and verify the results of the *G. mellonella* larvae infection models. Furthermore, this data can be combined with bacterial genome-wide association analyses to identify novel virulence factor gene candidates in this understudied bacterial species. *H. ovis* isolated from other hosts and body sites should also be evaluated with this model to look for virulence patterns across the species that may aid treatment and infection prevention.

## Materials and methods

### Sample collection and disease definition

All procedures involving cows were approved by the Institutional Animal Care and Use Committee of the University of Florida; protocol number 201,910,623.

A total of 5 lactating Holstein Friesian cows from the University of Florida’s Dairy Research Unit in north central Florida were used for this study. All cows had a uterine swab collected and had their uterine discharge evaluated at 4, 6, and 8 d postpartum. Uterine discharge was scored on a 5-point scale as previously described [27]: Score 1 = not fetid normal lochia, viscous, clear, red, or brown; 2 = cloudy mucoid discharge with flecks of pus; 3 = not fetid mucopurulent discharge with <50% pus; 4 = not fetid mucopurulent discharge with >50% pus; 5 = fetid red-brownish, watery discharge. Cows with uterine discharge scores of 1–4 were deemed healthy and those with a uterine discharge score of 5 were diagnosed as having metritis. Uterine discharge samples were collected from healthy cows and cows with metritis using a sterile polyester culture swab. Culture swabs were suspended BHI broth with 30% glycerol and stored at −80°C. Additional sample information is provided in the supplementary material.

### Bacteria isolation, selection, growth, and standardization

A 20µL of uterine discharge suspension was streaked onto *Helcococcus* selective agar and incubated for 72 h at 36°C in aerobic conditions with 6% CO2 [28]. Individual colonies were selected based on morphology, sub-cultured, and identified via 16S rRNA gene sequence analysis as previously described [29]. Two *H. ovis* isolates (KG36 and KG38) were selected for initial testing of the infection model. The stock inoculum for each isolate was grown in BHI broth supplemented with 0.1% Tween 80. At the time of inoculation, serial ten-fold dilutions were made with target doses of 10^8^ (high) to 10^2^ (low), with 10^6^ and 10^4^ colony forming units as intermediary doses. All infectious doses were confirmed by a quantitative culture of serial dilutions at the time of infection.

### *Maintenance and selection of* G. mellonella

The insects used in the bioassay were purchased from Vanderhorst Wholesale Inc. (OH, USA), a commercial insectary. At the time of arrival, hemolymph was extracted and cultured in BHI broth and blood agar (trypticase soy agar (TSA) with sheep blood) for quality control. Only last-instar larvae, greater than 2 cm in length, free of signs of trauma from transport, and without signs of melanization were used for the bioassay. Melanized and non-motile larvae were separated and discarded. Larvae deemed adequate for inclusion in the bioassay were stored in vented plastic containers with wood chips, at room temperature, for up to 5 d. No food was provided to the larvae.

#### G. mellonella inoculation with H. ovis *isolates*

For inoculate preparation, mid-log phase culture of each isolate in BHI broth with 0.1% Tween 80 was centrifuged at 3000 g for 10 min. The resulting supernatant was filtered through a 0.22-micron filter, cultured in BHI broth and blood agar (TSA with sheep blood and 0.01% pyridoxal HCl) for quality control. Bacterial pellets were washed twice in ready-made phosphate buffered saline (PBS, Fisher Scientific, Gibco™, Catalog# 70-011-044), and re-suspended in PBS to an OD_600_ measurement of 0.8 as a relative measure for 1 × 10^8^ CFU/ml. Serial dilutions were carried out and confirmed through serial dilution plating to achieve the target doses of 1 × 10^6^, 1 × 10^4^, and 1 × 10^2^ CFU. Injection groups with culture filtrates of KG36 and KG38 served as a control for potential suspended metabolites and exotoxins. An injection group of sterile PBS served as media injection controls. Lastly, a group of uninoculated larvae were used as environmental controls.

Inoculation was carried out via injection of 10uL of inoculum via the last-left proleg using 31-gauge single-use U100 insulin syringes with 0.5-unit markings (Becton, Dickinson and Company, USA). Infected larvae were individually placed into a well of a Costar 24-well cell culture plate (Corning, USA). Cell culture plates were then placed in an incubator at 37°C for the first validation attempt and then at 36°C and 5% humidity for the remaining bioassays.

For the initial validation of the model, 50 larvae were used per isolate per dose. This sample size was calculated to allow for the detection of a 25% difference in mortality, with a significance level of 5% and power of 80%. Once the bioassay was validated, virulence testing inoculation groups were reduced to 48 larvae each for housing convenience.

## Monitoring

For the initial validation of the bioassay, larvae were checked for morbidity, mortality, and pupation every 24 h for 14 d. Supplemental Figures 1 and 2 illustrate the automated time-lapse photography system was assembled to monitor the larvae for motility and melanization. The system consisted of a GoPro Hero4 camera (GoPro, USA), mounted onto the ceiling of the incubator, programmed to take a photograph once per minute for the duration of the experiment. An LED light strip was placed around the camera on a looping timer to turn on in synchrony with the photos being taken to reduce the larvae light exposure. Wax paper was placed between the light and the larvae as a light diffuser.

## Bacterial colonization

Larvae were injected with 1 × 10^7^ CFU of *H. ovis* KG36, 1 × 10^7^ CFU *H. ovis* KG38, or sterile PBS and incubated at 36°C. Three live larvae were randomly selected at 2-, 6-, 12-, and 24-h post-inoculation from each group for hemolymph extraction. Each selected larva was put inside a sterile petri dish and placed on ice for 5 min before hemolymph extraction. A sterile 18-gauge needle was used to make a puncture between the last four prolegs and 10 µL of the extruded hemolymph was collected with an Eppendorf pipette. Serial tenfold dilutions were done from extracted hemolymph in chilled sterile BHI broth, plated on Columbia blood agar with 0.002% pyridoxal HCl, and incubated at 36°C, 5% humidity, and 6% CO2 for 76 h for CFU quantitation.

## Histology

Larvae were injected with 1 × 10^7^ CFU of *H. ovis* KG36 or with PBS and incubated at 36°C. Live larvae were selected at 2-, 6-, and 24-h post-inoculation, separated, injected with 50uL of ready-made 10% buffered formalin (Fisher Scientific, Cat# SF98–20), pH 6.9–7.1, and stored in 10 mL of 10% buffered formalin at 4°C for 5 d. Fixed larvae were cut along the sagittal plane into two segments using a scalpel, embedded in paraffin, and sectioned at 5 μm using a microtome. Sections were stained using hematoxylin and eosin and Gram stain. Hemocytes were identified as previously reported [30]. Supplemental Figure 4B depicts a prohemocyte and a plasmatocyte. Histological procedures were performed at the University of Florida College of Veterinary Medicine Anatomic Pathology Service.

## Statistical analysis

Differences in virulence were analyzed using the Kaplan–Meier survival analysis with the log-rank significance test to compare survival curves among doses and isolates. Bonferroni corrected/adjusted *p* values were used in cases where multiple log-rank significance test comparisons were performed. The bacterial colonization of hemolymph raw data was transformed to their base-10 logarithm to achieve normality. Differences in microbial load in the hemolymph between KG36 and KG38 was compared at each time point using one-way ANOVA. Differences in microbial load in the hemolymph at 2, 6, 12, and 24 h were compared using one-way ANOVA followed by multiple comparison test with Tukey’s correction for multiple comparisons. All statistical analyses were performed using GraphPad Prism version 9.4.1 for Windows, GraphPad Software, San Diego, California, USA. Differences with P ≤ 0.05 were considered significant.

## Supplementary Material

Supplemental Material

## Data Availability

The datasets generated during the current study are available as supplementary files.

## Highlights

- *Helcococcus ovis* proliferated in the hemolymph and induced dose-dependent mortality in *Galleria mellonella.*
- *Helcococcus ovis* isolates with high, medium, and low virulence were identified.
- The model had a quick turnaround, with differences in induced mortality between *Helcococcus ovis* isolates of only 48 h.
- *G. mellonella* employed hemocyte-mediated immune responses to *H. ovis* infection, analogous to the innate immune response in cows.
- *Galleria mellonella* can be used as an invertebrate infection model for the emerging multi-host pathogen *Helcococcus ovis.*
