# Supplementary figures and images for: Establishing Galleria mellonella as an invertebrate model for the emerging multi-host pathogen Helcococcus ovis
Source: Virulence. 2023 Mar 12;14(1):2186377. doi: 10.1080/21505594.2023.2186377 (PMC10026881; doi:10.1080/21505594.2023.2186377)

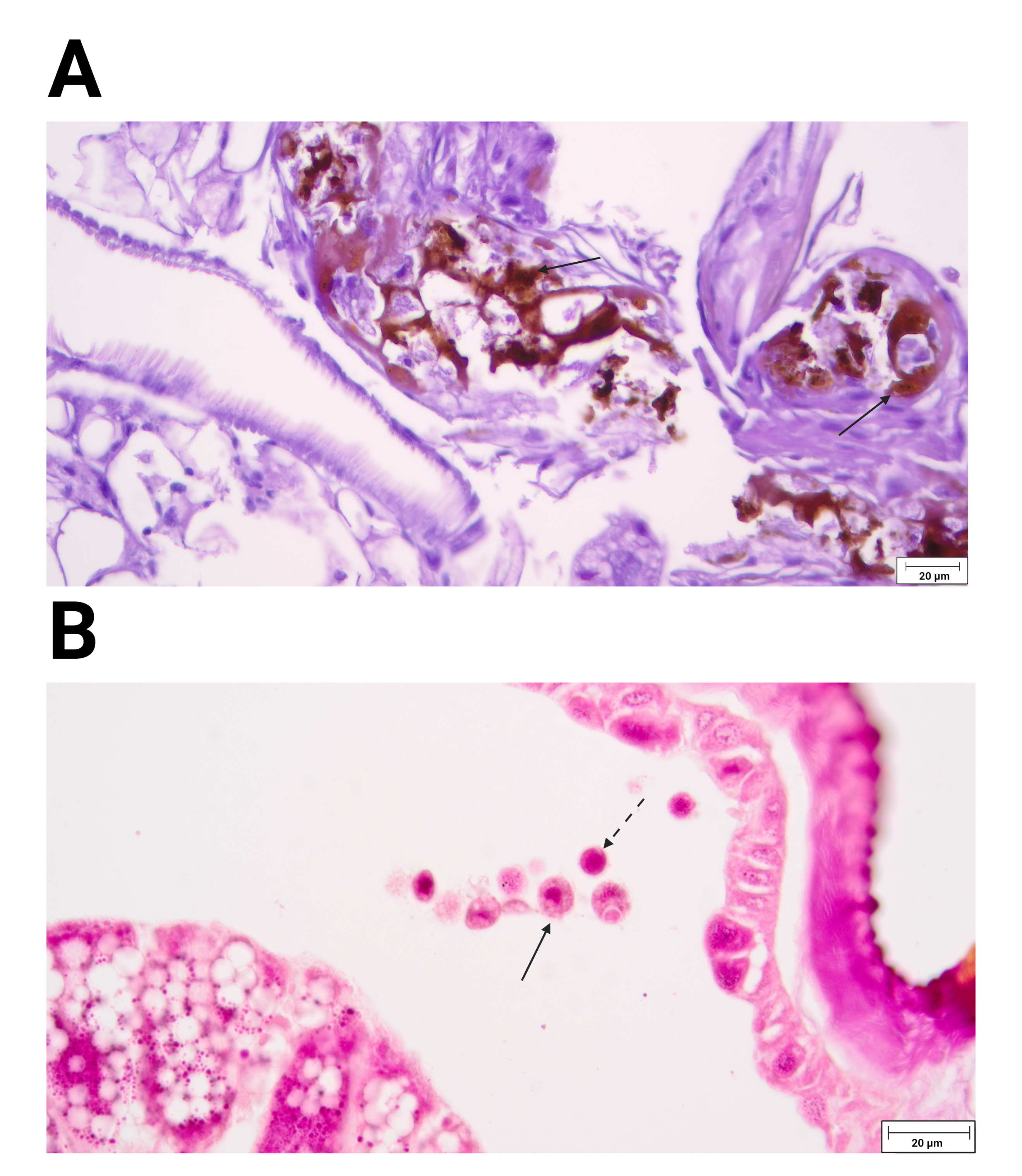

Supplement: Supplemental Material [file KVIR_A_2186377_SM1013.zip › Suplemental Figure 4.png]

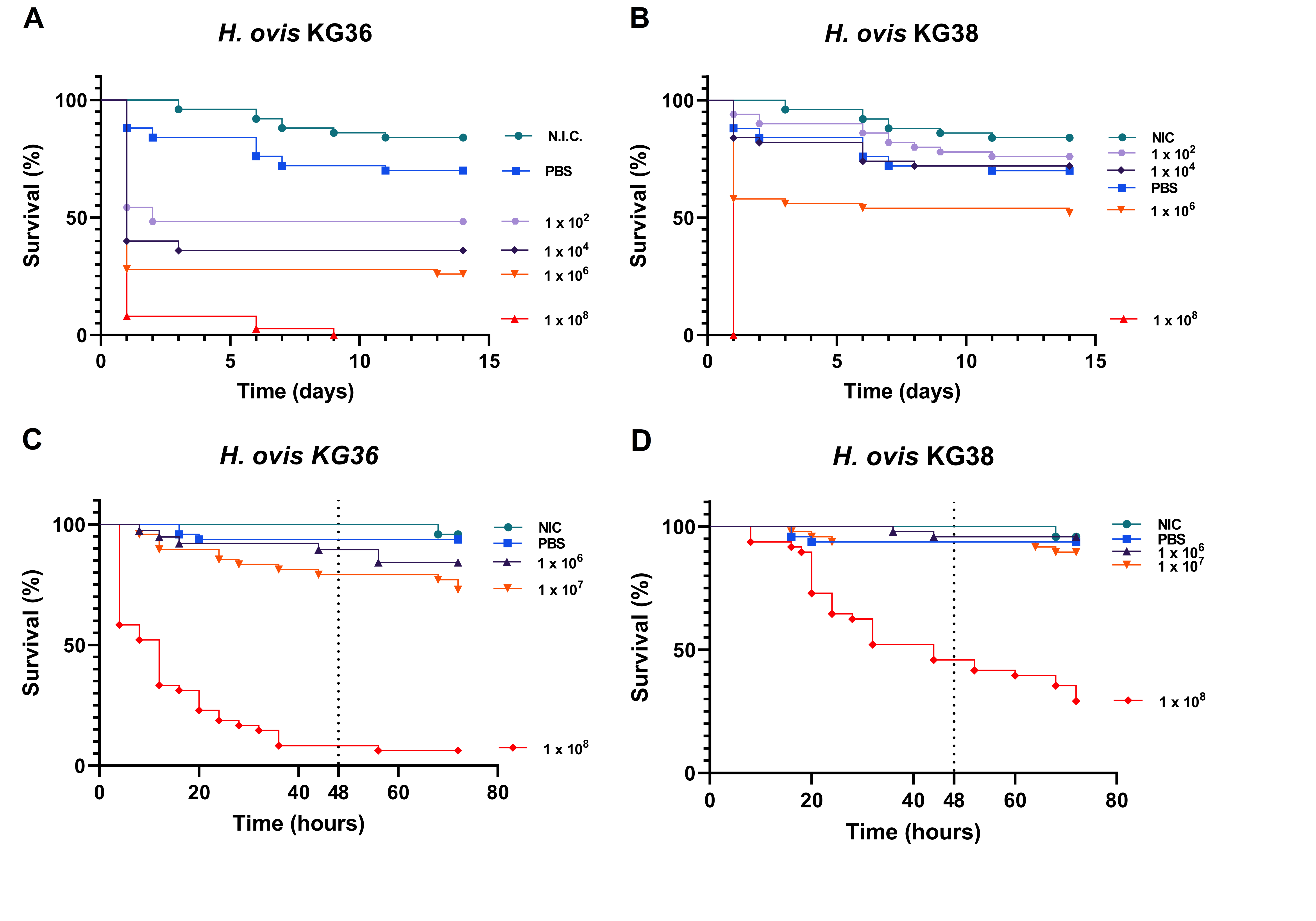

Supplement: Supplemental Material [file KVIR_A_2186377_SM1013.zip › Supplemental Figure 1.png]

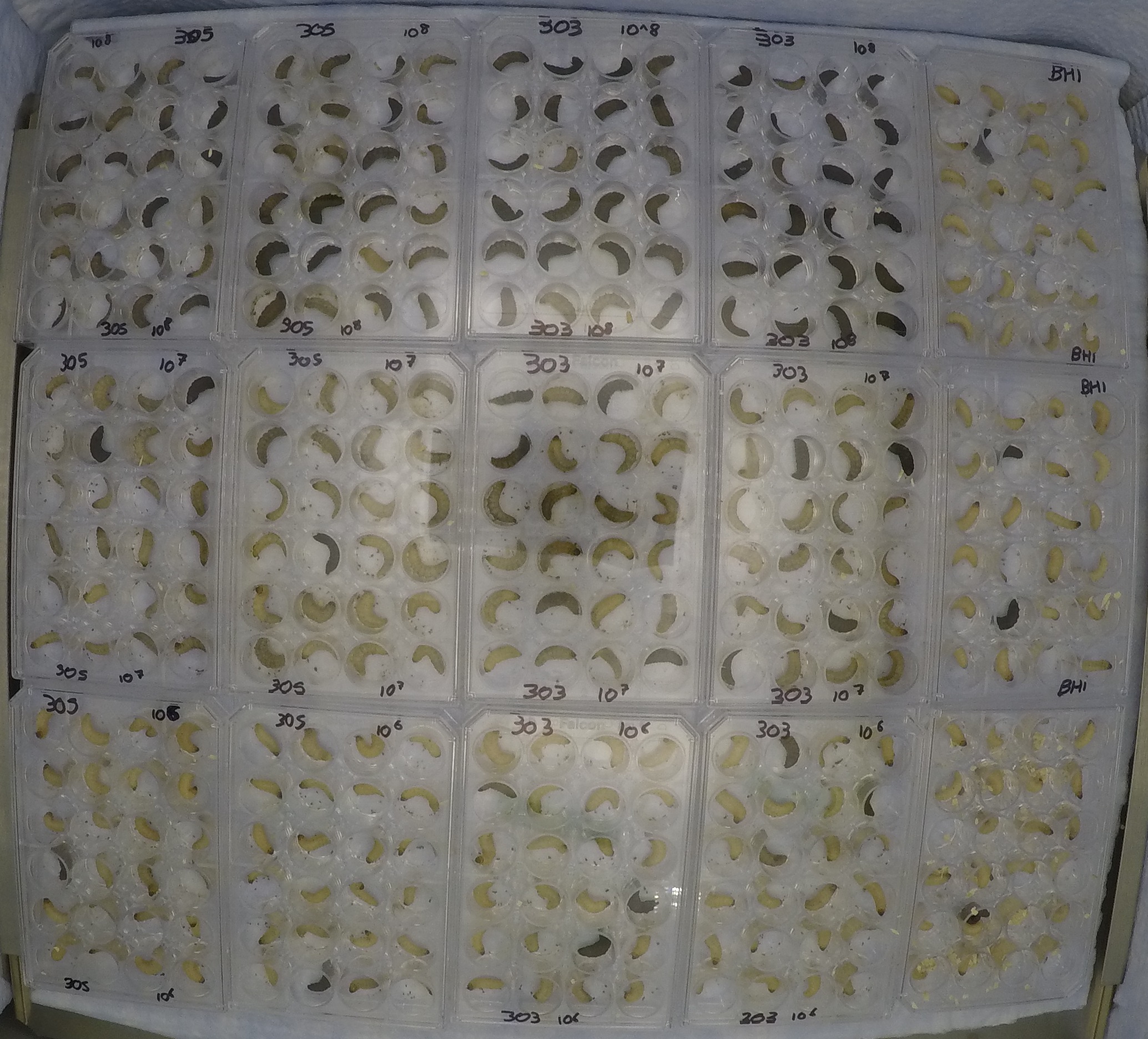

Supplement: Supplemental Material [file KVIR_A_2186377_SM1013.zip › Supplemental Figure 2.JPG]

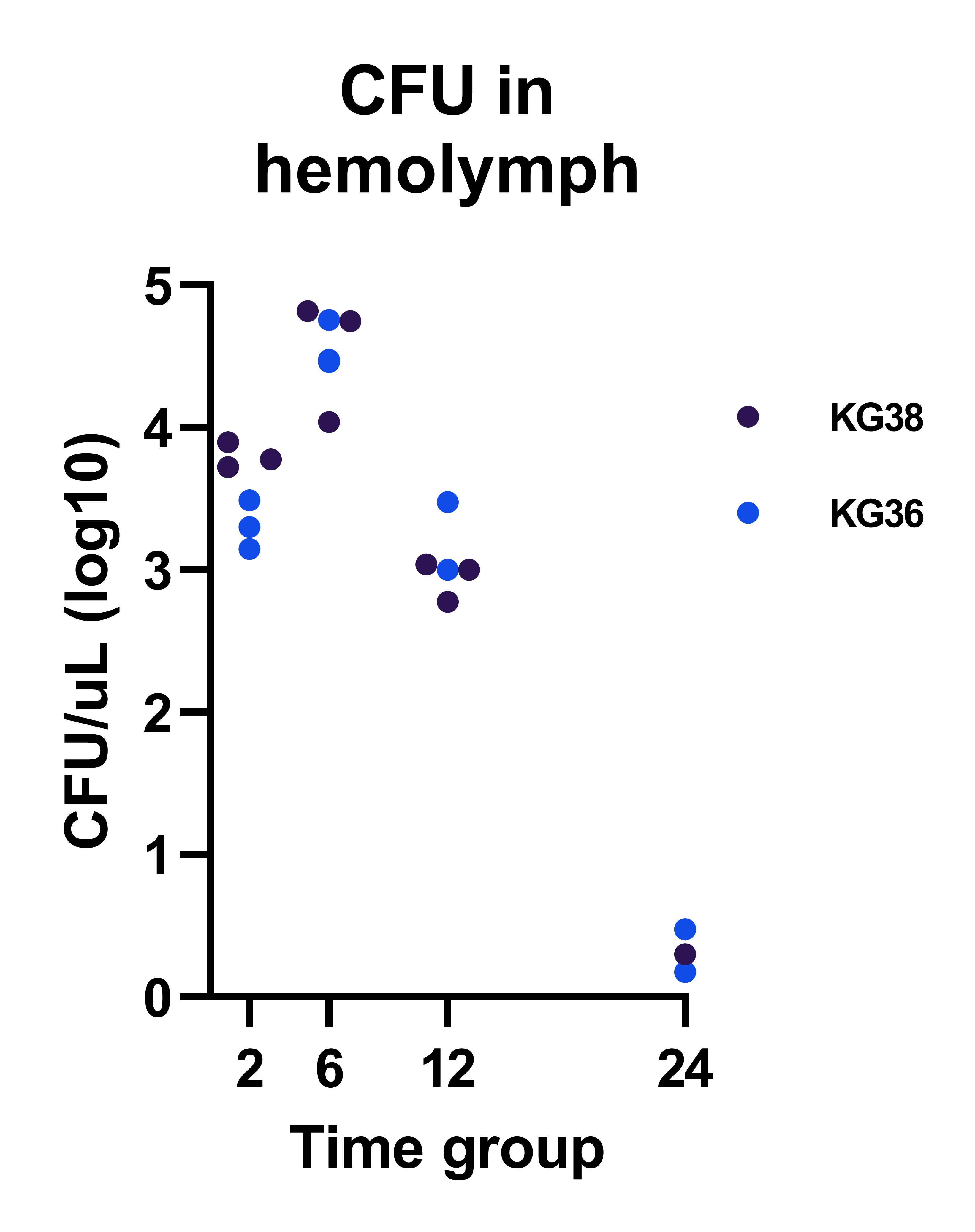

Supplement: Supplemental Material [file KVIR_A_2186377_SM1013.zip › Supplemental Figure 3.tif]
